# Effect of Methionine Restriction on Bone Density and NK Cell Activity

**DOI:** 10.1155/2016/3571810

**Published:** 2016-11-02

**Authors:** Mingxin Li, Lidong Zhai, Wanfu Wei, Jingming Dong

**Affiliations:** ^1^Tianjin Hospital, Jiefangnan Road 406, Tianjin 300210, China; ^2^Department of Anatomy and Histology, Basic Medical College, Tianjin Medical University, Tianjin 300070, China

## Abstract

Methionine restriction (MR) is proven to increase the lifespan; and it also affects the bone density and the innate immune system. The aim of this study is to explore the effect of methionine restriction on bone density and natural killer (NK) cells. C57BL/6J mice were subjected to either basal diet (BD, containing 0.80% methionine) or methionine-restricted diet (containing 0.14% methionine). Mice with MR diet displayed reduced bone mass and decrease in the cytotoxicity of NK from the spleen, compared to BD animals. Also, mice with MR diet had an inferior body weight (*P* < 0.05) and higher plasma levels of adiponectin and FGF21 (*P* < 0.05) but lower concentrations of leptin and IGF-1 (*P* < 0.05). Overall, the investigation shows that methionine affects bone density and NK cell cytotoxicity.

## 1. Introduction

In modern times, a person's health and fitness have become an important factor in everyday life, and researchers are pursuing ideas that enable people to improve their vitality and prolong their life. Experiments occurring in the last thirty years have used the restriction of certain macronutrients to promote a healthier lifestyle. While methionine restriction (MR) is a new invention to improve lifespan and vitality, protein restriction (PR) has been used for a lengthier period [1]. Also, caloric restriction (CR) has been a positive source to extend the average lifespan, plus increasing the maximal age [2]. This important and significant increase in lifespan can be principally accounted for due to age-related illnesses appearing later than in previous generations, plus the downregulation of oxidative stress [3]. Also, when dietary methionine content is restricted, energy metabolism is altered [4], and it can increase a rodent's lifetime by thirty percent [5]. This alone is interesting, but MR can also intensify concentrations of glutathione, which is an antioxidant, in blood. Also MR can lessen visceral adiposity and induce insulin receptiveness [3, 5].

There is a worry with MR that it causes reduced bone strength. This side-effect was verified in rats given MR resulting in stunted growth, making them light and diminutive [6]. MR could have an impact on bone growth/development, tissue material properties, and bone metabolism when related to alternative diets which use dietary restrictions. This idea comes from the fact that the consumption of MR, by rats, for a lengthened period of time results in the intake of more sustenance per unit of body weight (BW), when related to rats in control groups.

Gene expression can go through alterations if DNA-methylation patterns, facilitated by* S*-adenosylmethionine, when subjected to methionine-deficient diets [7]. Androgen-independent prostate cancer cells, when exposed to methionine created apoptosis, signify affected motility [8]; plus methionine reduces mitochondrial oxidative stress [9]. As we become older, methylation deteriorates, which can result in the reduction of T cells and NK cells. Unfortunately, this can alter the actions of the immune system, but methylation can reverse this and prevent this by assisting in the construction of natural killer (NK) cells. These cells signify a distinct lymphocyte subset, playing an important part in innate immunity. Findings in humans and mice propose that NK cells are a significant tool for influencing the functions of the adaptive immune response [10] and, due to their cytotoxic role, NK cells can fight against pathogens and tumors, in addition to other vital inception [11]. Thus, in this study, we examined correlation between MR and bone density and the cytotoxic activity of NK cells in C57BL/6J mice.

## 2. Material and Methods

### 2.1. Animal Care

During this project, procedures set out by the Laboratory Animal Ethical Commission of Tianjin Hospital were followed. In the beginning, we obtained, from the Tianjin Laboratory Animal Center (Tianjin, China), forty-seven-week-old C57BL/6J mice and accommodated them in typical housing, which was regulated at 22 ± 3°C and 53 ± 10% comparative humidity. They were given nourishment and water (pH 2.8) as needed and kept in a light cycle of twelve hours of light, followed by twelve hours of a dark photoperiod. When the fourteen-week experiment began, the mice were randomly divided into two groups, with one set given the basal diet (BD) consisting of 0.80% methionine and the other group receiving the methionine-restricted sustenance, consisting of 0.14% methionine. The researchers measured the body mass and the food intake of the mice, twice per week, with the average daily food eaten by every mouse being calculated to find the cumulative food amount. When the experiment drew to a climax, the mice were subjected to a four-hour fasting period, starting at the introduction of the light cycle. This was to assign a physiological baseline, before being sacrificed. After that, blood was obtained via the retroorbital plexus, a sample of plasma was flash-frozen and kept at −80°C, and femur and spleen bones were attained, flash-frozen, again at −80°C, and saved for examination.

During the study, enzyme-linked immunosorbent assay (ELISA) kits were utilized when recording the amount of apo B, leptin, adiponectin, insulin, homocysteine, IGF-1, and FGF21. Also, an Abbott® Freestyle glucometer and glucose strips were the choice to detect the amount of blood glucose. Colorimetric assays were needed to detect plasma triglycerides (TG), total cholesterol (TC), LDL, and HDL. Finally, a Beckman Synchron CX5 system distinguished the quantity of plasma alanine aminotransferase.

### 2.2. Preparation of Target Cells and NK Cell Degranulation and Cytotoxicity

Total RPMI 1640 medium, primed as stated above, was used to cultivate YAC-1 cells, which are murine T-lymphoma cell lines, receptive to NK cell killing. The habitat was kept at 37°C in 5% CO_2_, and cells were subcultured once a day to guarantee that the log-phase was displayed. During the essay, the cells were coloured with 10 nM of carboxyfluorescein succinimidyl ester in darkness for ten minutes. Then, they were cleaned thoroughly twice in PBS with 2% heat-inactivated fetal bovine serum, before being subjected to another solution of 10^6^ cells/mL in medium. The methods of preparation of NK cells and the cytotoxicity of these cells were gained during the flow cytometric assay defined by Cao et al. [12].

### 2.3. Statistical Analyses

The information is displayed as the means ± the standard error of the mean (SEM), and the statistical research was collated and scrutinised, by use of a SPSS 25.0 (Chicago, IL, USA). The significant differences between the sets, recorded by a Student's *t*-test, were defined at *P* < 0.05.

## 3. Results

**Figure 1:**
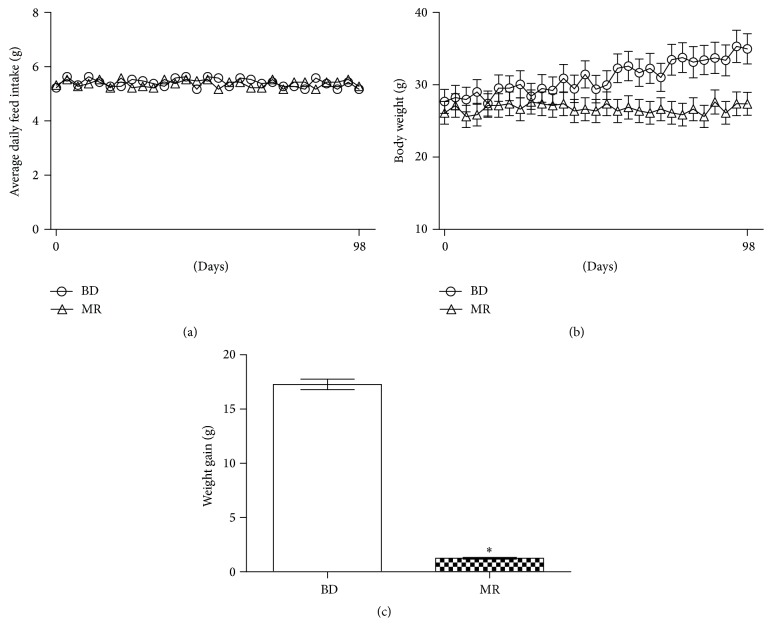
MR mice have lower body weight and lower body weight gain. (a) Average daily feed intake for 14 weeks. (b) Body weight was measured twice a week for 14 weeks. (c) Body weight gain. ^*∗*^ *P* < 0.05. *n* = 10.

To test whether MR diet can inhibit body weight gain, average daily food intake, body weight, and body weight gain were measured. As shown in Figure 1, average daily food intake was almost the same in both BD and MR mice during the whole period (Figure 1(a)). However, significantly lower body weight was found in MR mice compared to that in the BD group (*P* < 0.05) from three weeks after the start of the experiment (Figure 1(b)). Meanwhile, in the MR mice, a significantly lower weight gain (*P* < 0.05) was observed (Figure 1(c)). Data of plasma lipids indicated TG level was significantly lower in MR mice compared to that of BD mice (*P* < 0.05). Meanwhile, the levels of TC, HDL, LDL, and apo B were not altered in both groups (Table 1). As represented in Table 1, the study of plasma levels of hormones linked to the resistance of insulin was carried out. Mice with MR diet had higher (*P* < 0.05) level of adiponectin and FGF21 but lower concentration of leptin and IGF-1 (*P* < 0.05).

Table 2 confirmed the concern that an MR diet altered the density and structure of bones, as investigations into bone consistency revealed that MR mice were not as long as the BD mice (*P* < 0.05). Also, MR animals had left femurs which were diminished when compared to BD mice (*P* < 0.05). Supplementary evidence comes in the form of the MR group displaying diminished amounts of BMC and BMD (*P* < 0.05), in a bone mineral density examination, by means of DEXA. Also, in contrast to BD mice, MR animals had slighter diameters of mediolateral and anteroposterior shafts, and the third trochanter shafts were also prominently diminished (*P* < 0.05).

**Figure 2:**
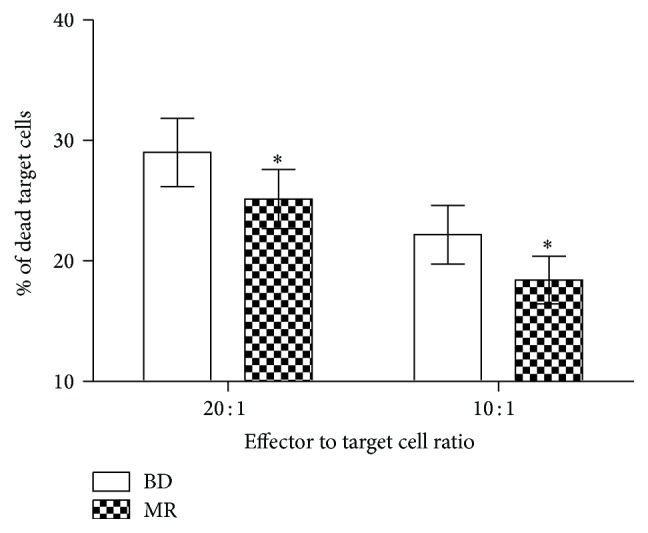
The consequence of the MR diet on NK cell cytotoxicity. Splenocytes were secluded before being gestated with YAC-1 target cells at 20 : 1 and 10 : 1 ratio of effector to target cells. Dead target cells were enumerated by flow cytometry, and the quantities are presented as means ± SEM, *n* = 8. ^*∗*^ *P* < 0.05.

**Figure 3:**
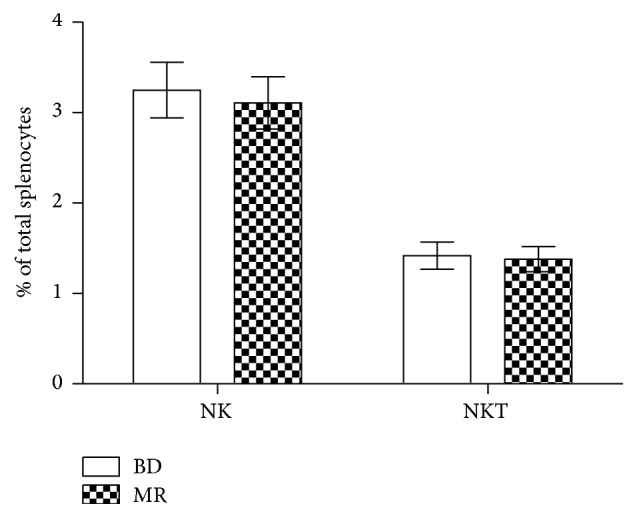
The influence the MR diet has on aged mice within NK cells. Splenocytes are recorded as percent of NK or NKT cells in entire splenocytes enumerated. Data are presented as means ± SEM, *n* = 8.

Another area which is found to be pointedly lessened in MR animals, in contrast to BD mice, is NK cell cytotoxicity, which has cell ratios of 20 : 1 for MR mice and 10 : 1 in BD animals (Figure 2). The NK cell cytotoxicity, consuming BD diet or MR diet, is so diverse; this is because the cytotoxicity assay records the action of both NK and NKT cells. As shown in Figure 3, the spleens of both diet sets recorded a similar percentage of NK and NKT cells (*P* > 0.05).

## 4. Discussion

In this experiment, methionine was restricted in, not eradicated from, the diet of the mice, similarly to other MR examinations, where the experiments were performed on rats, and proved to prolong their lifespan [6, 13]. Furthermore, the restrictive consumption of methionine decreased mitochondrial reactive oxygen species in rats [14] indicated that their levels of lipogenic, lipolytic, and conceivably hypercholesterolemia progressed [15]. When witnessing the rats, we were conscious to note that the rodents did not suffer any ill effects while being subjected to the MR diet.

In this experiment, comparisons between the different sets of mice were varied. The typical daily food consumption was comparable in MR and BD groups, while the animals in the MR set displayed a pointedly diminished body weight when contrasted, three weeks into the study and thereafter, with the BD group. Moreover, there was a connection found with the reduction of bone density. In contrast, MR mice, which were given high fat diet, signified a reduction in body weight gain, albeit developing hyperphagia, which is known to occur in rats and mice, consuming low-fat foods [13]. The hyperphagia could be seen in the animals because of their bodies producing elevated levels of glutamic acid, in a need to compensate for the decline in methionine consistencies [16]. However, ultimately, the movement and physical planes of the rats consuming a reduced methionine diet and the mice who were given the total required amount of amino acids were comparable.

The result of the MR diet found in the configuration of the C57BL/6J mice was comparable with plasma adiponectin, which had elevated levels, and also with the diminished quantities of IGF-1, leptin, and insulin [4]. Heightened levels of adiponectin were recorded in the mice in the MR group, and these levels have been known to potentiate insulin receptive properties, via the initiation of PPAR*γ* signaling [17]. FGF21 is a hormone, newly identified and capable of immense interaction with glucose homeostasis [18]. The MR mice, in this study, indicated heightened planes of FGF21, within their plasma, when compared to BD grouped animals, and when analysed further, MR mice show a similar hormone make-up to those of insulin sensitive animals. FGF21, when subjected to MR, can clarify, to some extent, the reductions in growth witnessed in the MR animals [19]. There are surprising correlations between the research carried out in this study and alternative explorations, such as the interpretations of Inagaki and company, where FGF21 transgenic animals displayed not only a smaller mass, but also declined amounts of plasma IGF-1 in comparison to the wild-type versions [20]. Furthermore, these researchers found that the influence of FGF21 was moderated by phosphorylated Stat5 being heightened and Jak2 phosphorylation being diminished [20]. This brings about an interesting point concerning our data, in the context of FGF21 being the agent which disturbs the growth hormone signaling downstream of Jak2. In this alternative study, wild-type mice suffered augmented bone deterioration, by means of the PPAR*γ* agonist, rosiglitazone, whereas no change was recorded in the FGF21 knockout mice, possibly concluding that FGF21 could be the attribute responsible for diminished bone density in PPAR*γ* signaling [21]. Therefore, it is plausible that the lessening of bone mass in MR mice could be happening due to heightened FGF21 involvement.

The MR diet's influence on NK cytotoxicity was then further investigated, with no major variances in NK cells percentage amongst dietary sets. NK cell cytotoxicity and immune activity benefit from receiving satisfactory methionine, and this project has proved that methionine restriction is related to reduced NK cell function. For these years, there have been ongoing studies into the correlations between an intensified risk of cancer and reduced NK cytotoxicity [22]. The results indicated a reduced amount of NK cell cytotoxicity, because of restricted methionine, increased the risk of cancer and viral infections, particularly in older generations.

In conclusion, the animals subjected to the MR diet did not display any unhealthy side-effects except the inferior body weight and lower bone density. The accumulation of information concerning the study exhibited reduced NK cell cytotoxicity, which could be due to weakening or damage to NK cells while maturing. Additional studies are needed regarding the impact made on NK cell cytotoxicity by restricted methionine and the effects for improving health in humans.

## Figures and Tables

**Table 1:** Plasma biochemistry of BD mice and MR mice (*n* = 8, ^*∗*^ *P* < 0.05).

	BD group	MR group
TG (mg/dL)	67.25 ± 8.12	55.78 ± 4.86^*∗*^
TC (mg/dL)	145.83 ± 12.47	149.32 ± 13.27
LDL (mg/dL)	32.76 ± 2.89	33.14 ± 2.43
HDL (mg/dL)	106.37 ± 9.54	105.38 ± 8.89
Apo B (*μ*g/dL)	30.1 ± 3.5	30.9 ± 3.3
Adiponectin (ng/dL)	3.67 ± 0.42	5.78 ± 0.76^*∗*^
FGF21 (pg/mL)	78.76 ± 7.12	97.48 ± 10.54^*∗*^
IGF-1 (pg/mL)	424.85 ± 54.18	281.24 ± 31.19^*∗*^
Leptin (pg/mL)	210.11 ± 23.42	120.56 ± 14.55^*∗*^

**Table 2:** Bone parameters of femurs from BD mice and MR mice (*n* = 8, ^*∗*^ *P* < 0.05).

	BD group	MR group
Femur length (mm)	16.23 ± 0.54	15.43 ± 0.48^*∗*^
Mediolateral shaft diameter (mm)	2.12 ± 0.13	1.91 ± 0.13^*∗*^
Anteroposterior shaft diameter (mm)	1.36 ± 0.11	1.21 ± 0.08^*∗*^
Third trochanter diameter (mm)	2.85 ± 0.17	2.53 ± 0.28^*∗*^
BMD (g/cm^2^)	0.061 ± 0.008	0.049 ± 0.006^*∗*^
BMC (g)	0.031 ± 0.006	0.024 ± 0.005^*∗*^
